# Non-ovarian *Wolbachia pipientis* titer correlates with fertility rescue of a *Drosophila melanogaster bag of marbles* hypomorph

**DOI:** 10.17912/micropub.biology.001233

**Published:** 2024-08-02

**Authors:** Catherine H. Kagemann, Gabriela M. Colocho, Charles F. Aquadro

**Affiliations:** 1 Molecular Biology and Genetics, Cornell University, Ithaca, New York, United States

## Abstract

*Bag of marbles* ( *bam* ) is an essential gene that regulates germline stem cell maintenance and germline stem cell daughter cell differentiation in *Drosophila melanogaster* . When *bam* is partially functional (hypomorphic), the introduction of *Wolbachia pipientis* rescues the mutant fertility phenotype that would otherwise result in partial sterility. Infection by different *W. pipientis* variants results in differential rescue of the *bam* hypomorph fertility phenotype. We were intrigued by the varying degrees of rescue exhibited in the *bam* hypomorph when exposed to different *W. pipientis* variants, prompting us to investigate whether this phenomenon is attributable to variations in the titers of *W. pipientis* variants. We found no significant difference in ovarian titer between two *W. pipientis* variant groups, *w* Mel-like (low *bam* hypomorph fertility rescue) and *w* MelCS-like variants (higher *bam* hypomorph fertility rescue), at *bam* hypomorph peak fertility. However, carcass (whole flies without the ovaries) titer between *w* Mel-like and *w* MelCS-like infected *bam* hypomorph differed during peak fertility rescue. A positive correlation emerged between the combined titers of ovarian and carcass infections and fertility, implying a more extensive influence that extends beyond ovarian infection alone.

**Figure 1. Absolute quantification of W. pipientis titers in female flies shows that both ovarian and carcass W. pipientis titer have a positive correlation with fertility.**
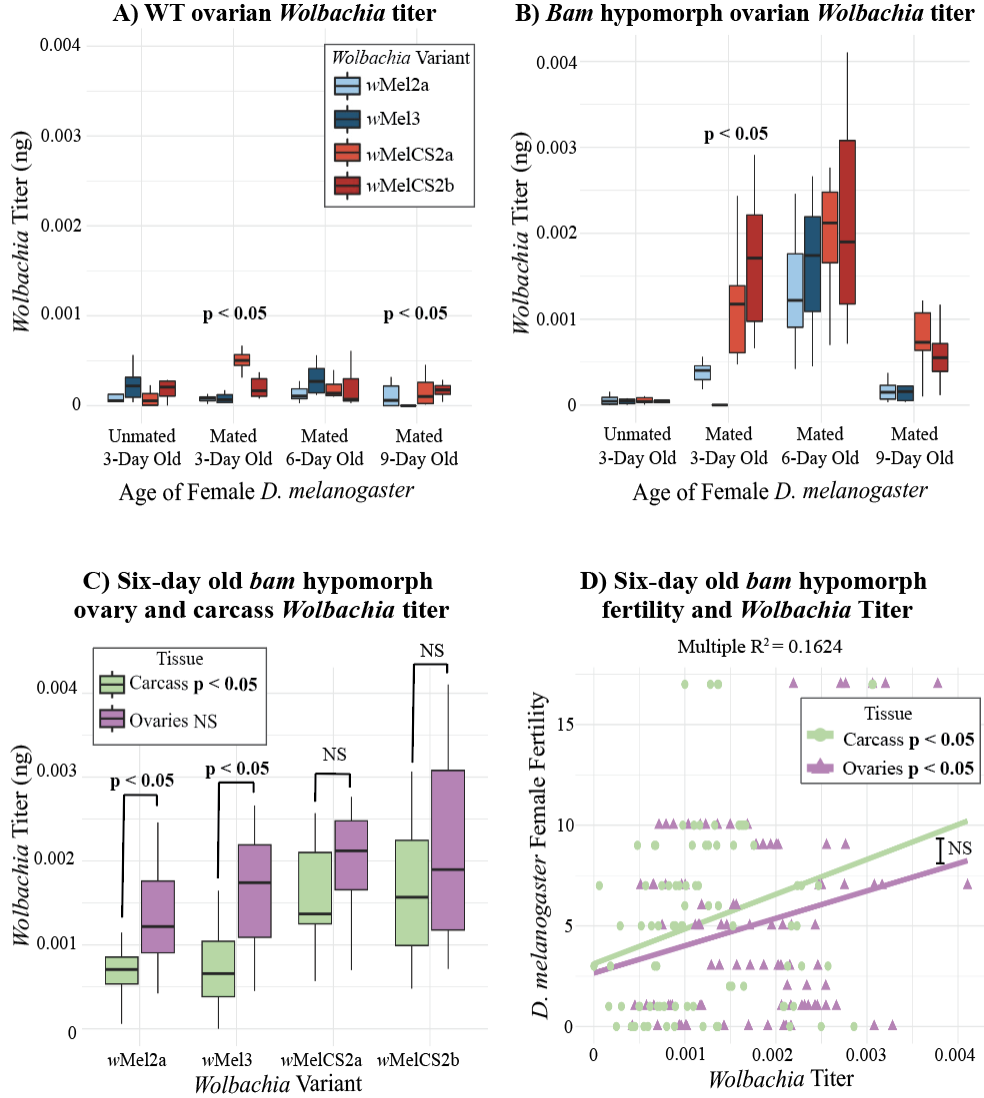
*W. pipientis* titer in female (A) WT and (B) *bam* hypomorph *D. melanogaster* ovaries calculated using absolute quantification qPCR. P < 0.05 is specified at a given age/mating status if there is a significant difference between *w* Mel-like and *w* MelCS-like variant titer (linear regression model, R). (C) Ovarian and carcass *W. pipientis* titer during *bam* hypomorph peak fertility rescue (six-day old). P < 0.05 in the legend designates that there is a significant difference in *w* Mel-like and *w* MelCS-like *W. pipientis* carcass titer while there are no significant differences (NS = Not significant) in the ovaries (linear regression model, R). P < 0.05 or NS is indicated between carcass and ovarian *W. pipientis* variant titer (Two-Way ANOVA, R). (D) The correlation between *D. melanogaster* fertility and *W. pipientis* (all variant titer combined) ovarian and carcass titer. P < 0.05 is indicated in the legend to represent a statistically significant correlation between fertility and *W. pipientis* in both the ovaries and carcass (linear regression model, R). NS between the linear regression lines indicates no statistically significant difference between slopes.

## Description

Bag of marbles (Bam) is a protein necessary for oogenesis and spermatogenesis in *Drosophila melanogaster* (McKearin and Spradling 1990) *.* Bam triggers germline stem cell (GSC) daughter cell differentiation during *D. melanogaster* oogenesis and regulated GSC maintenance (McKearin and Spradling 1990) . It was previously determined that the introduction of a maternally inherited bacterial endosymbiont, *Wolbachia pipientis,* rescues the mutant fertility phenotype in *bam* partial loss-of-function (hypomorph) mutants (Flores et al. 2015) . Without *W. pipienti* s, *bam* hypomorph flies are partially sterile (Flores et al. 2015) . The interaction between *Wolbachia pipientis* and the *bam* hypomorph prompted us to study the mechanisms that could play a role in the mutant fertility rescue.

*W. pipientis* , exhibits a diversity of variants, including well-known variants like *w* Mel, *w* Yak, and *w* Ri (Riegler et al. 2005) . Among these, the *w* Mel strain, infecting *D. melanogaster* , displays various variants (e.g. *w* Mel2a, *w* Mel3, *w* MelCS2a, and *w* MelCS2b) each influencing distinct host phenotypes (Bubnell et al. 2021; Christensen et al. 2019; Chrostek et al. 2013; Truitt et al. 2019) . These phenotypes encompass characteristics such as the intensity of cytoplasmic incompatibility, protection against viruses, and the host's temperature preference (Bubnell et al. 2021; Christensen et al. 2019; Chrostek et al. 2013; Truitt et al. 2019) .

There is a correlation between *W. pipienti* s titer and variant genotype that results in specific host phenotypes (Chrostek et al. 2013; Ilinsky and Zakharov 2011) . For example, *w* Mel-like variants which have a lower titer, confer lower viral protection but a longer host lifespan in males (Chrostek et al. 2013) . *W* MelCS-like variants cause higher viral protection and a shorter lifespan in male *D. melanogaster* (Chrostek et al. 2013) . Bubnell et al. (2022) determined that *w* MelCS-like infection in the *bam* hypomorph results in higher mutant fertility rescue than flies infected with *w* Mel-like variants. As there is evidence that *Wolbachia* genotype and titer increase specific host phenotypes, we questioned whether there is an association between *W. pipientis* titer and the differential *bam* hypomorph fertility rescue.

Kagemann et al. (2023) determined that there is a general correlation between ovarian *W. pipientis* titer and increases in *bam* hypomorph rescue. However, they did not address whether the differential rescue of *bam* hypomorph by different *W. pipientis* variants was driven by differences in *W. pipientis* variant titer or by other mechanisms specific to each variant (Kagemann et al. 2023) . While an increased *W. pipientis* titer alone may not necessarily lead to a direct increased fertility rescue in the *bam* hypomorph, an elevated titer, particularly influenced by specific strains of *W. pipientis* , has the potential to broaden the spectrum of host modifications. This, in turn, may enhance the effectiveness of fertility rescue in the *bam* hypomorph, especially when infected with W. *pipientis* variants.

*W. pipienti* s titer has traditionally been measured using relative qPCR which utilizes the ratio of a *W. pipienti* s-specific gene, such as *wsp* or *arm* , relative to a host gene, such as *rpl32* in *D. melanogaster* (Christensen et al. 2019; Cogni et al. 2021; Gnainsky et al. 2021; Kagemann et al. 2023) . However, relative qPCR assumes that host gene copy number is stable across conditions that are tested (Christensen et al. 2019) . Absolute quantification qPCR of *W. pipienti* s titer overcomes the limitations associated with relative quantification (Christensen et al. 2019) . This is achieved by comparing the copy number of *W. pipienti* s samples to a standard curve, allowing for the extrapolation of the total *W. pipienti* s copy number. As the *W. pipientis* infected *bam* hypomorph contains a mix of over proliferating GSC-like cells and WT GSC daughter cells, it is important to use absolute quantification to measure titer. This necessity arises from the potential variation in host gene copy numbers attributable to the diverse cell types present in the population. Our study is the first to use absolute quantification to measure ovarian and carcass *W. pipientis* titer of *w* Mel2a, *w* Mel3, *w* MelCS2a, and *w* MelCS2b variants infecting wildtype *D. melanogaster* and *bam* hypomorph genotypes of different ages/mating statuses.

Our results show that ovarian titer of all *W. pipientis* variants remained low as the female, wildtype flies aged, which contrasts with the increase that has been reported in whole male flies ( Fig. 1A ). The *w* MelCS-like variants infecting wildtype *D. melanogaster* had a higher ovarian titer than the *w* Mel-like variants infecting *D. melanogaster* in mated three-day old and nine-day old flies, but not in unmated three-day old or mated six-day old flies ( Fig. 1A linear regression model, p < 0.05).

Individual *W. pipientis* variant titer revealed that *bam* hypomorph flies infected with the *w* MelCS-like ( *w* MelCS2a and *w* MelCS2b) variants had a significantly higher titer than flies infected with the *w* Mel-like ( *w* Mel2a and *w* Mel3) variants in three and nine-day old flies, but not in six-day old flies ( Fig. 1B, linear regression model, p < 0.05). Kagemann et al. (2023) observed that the largest increase in *bam* hypomorph fertility rescue occurred in six-day old flies (peak fertility). However, we did not observe a statistically significant difference in ovarian titer between the *W. pipientis* groups ( *w* Mel-like and *w* MelCS-like) in six-day old flies suggesting another factor drives the difference in fertility rescue. We then asked whether *bam* hypomorph fertility rescue could be influenced by *W. pipientis* present in tissues outside of the ovaries during peak fertility rescue. To do this, we repeated the experiment using ovaries and carcass (whole flies excluding ovaries) from six-day old female *bam* hypomorph flies and discovered that the *w* MelCS-like variant carcass titer is significantly higher than the *w* Mel-like variant carcass titer ( Fig. 1C, linear regression model, p < 0.05). *W* Mel-like variant titer in the ovaries is higher than in the carcass (ANOVA, R).

We measured the fertility of the parent female flies in which ovary and carcass titer were measured in six-day old *bam* hypomorph flies. Due to our sample sizes (N=5 for each *W. pipientis* variant), we were unable to make comparisons between *w* Mel-like or *w* MelCS-like variant titer and fertility. Our correlation analyses between fertility and combined *W. pipientis* (all variants) titer show a positive correlation between both ovarian and carcass titer and fertility (Fig 1D). While the correlation between carcass titer and fertility seems higher than the correlation between ovarian titer and fertility, there is not a statistically significant difference (linear regression mode, p > 0.05, Fig 1D). With increased sample sizes, we hypothesize that *w* MelCS-like carcass titer would correlate with higher *bam* hypomorph fertility rescue compared to *w* Mel-like variant infected *bam* hypomorph flies.

There are at least two possible mechanisms by which *W. pipientis* titer outside of the ovaries could be influencing *bam* hypomorph fertility rescue. *W. pipientis* have a type IV secretion system (T4SS) that allows proteins to move from cell to cell (Rancès et al. 2008). It is possible that the *W. pipientis* variant groups secrete different amounts of proteins, peptides, or RNA into the ovaries that could be contributing to differential rescue of the *bam* hypomorph by different *W. pipientis* variants. This is reminiscent of the impact of gut bacteria on *D. melanogaster oogenesis* (Gnainsky et al. 2021) . Secondly, the regulatory mechanisms governing *W. pipientis* titer in the carcass may dictate fertility rescue in the flies, drawing parallels with the interplay between Notch and Wnt signaling—crucial components in gametogenesis—and their involvement in immune cell differentiation (Rogan et al. 2019) .

Building upon existing knowledge of *W. pipientis* strain diversity and its impact on host phenotypes, our investigation, employing absolute quantification qPCR, provides novel insights into the ovarian and carcass titers of *w* Mel-like and *w* MelCS-like variants in WT and *bam* hypomorph *D. melanogaster.* While the observed differences in ovarian titer between these variants in specific age and mating conditions align with prior findings, our revelation of significantly higher carcass titers for *w* MelCS-like variants prompts further inquiry into their potential influence on fertility rescue. The positive correlation between combined ovarian and carcass titers and fertility suggests a broader impact beyond ovarian infection alone.

## Methods

**Fly Strains and Absolute Quantification of *W. pipientis* Titer**

The *bam* ^L255F^ hypomorph mutation we used was recently remade using the same single amino acid change as the original *bam* ^BW^ hypomorph but in a *w* ^1118^ isogenic background (Bubnell et al., 2021; Bubnell et al. 2022; Flores et al. 2015) . The female *bam* hypomorph ( *w* ^1118^ ; *bam* ^L255F^ / *bam* ^[3xP3dsRed])^ that we used was generated by crossing a *bam* ^L255F^ /TM6 female to a *bam* null ( *w* ^1118^ ; *bam* ^[3xP3dsRed]/^ TM6) male (McKearin and Spradling 1990; Bubnell et al 2021) . An uninfected *bam* hypomorph control was used along with WT *bam* *w* ^1118^ fly lines containing each *W. pipientis* variant. The four *W. pipientis* variants and uninfected control were generously provided by Luis Teixeira and are described in Chrostek et al. (2013). All *D. melanogaster* lines were maintained on yeast glucose food and placed in an incubator at 25°C with a 12-hour light-dark cycle.

*W. pipientis* titer was first measured in WT and *bam* hypomorph ovaries in unmated three-day old, mated three-day old, mated six-day old, and mated nine-day old flies and is described in Kagemann et al. (2023). Five biological replicates were used per *W. pipientis* variant infecting the WT or *bam* hypomorph along with three technical replicates per biological replicate. We did not make a direct comparison of ovarian titers between the *Bam* hypomorph and WT because their DNA extractions were performed on different 96-well plates and diluted separately.

Subsequently, the experiment was repeated in six-day old *bam* hypomorph flies to measure both ovary and carcass *W. pipientis* titer. Flies were dissected from flash frozen female parents from our fertility assays using absolute quantification qPCR. Methods for absolute quantification of *W. pipientis* in the ovaries and carcass (whole flies without ovaries) are described in Kagemann et al. (2023). QPCR was run on a QuantStudio 7 Pro provided by Cornell’s Biotechnology Resource Center.

**Six-Day Old *Bam* Hypomorph Fertility Assay**

Our experimental design allowed us to quantify fertility and titer from the same *bam* hypomorph female flies (Fig 1D). We aged female *bam* hypomorph flies infected with each *W. pipientis* variant for five days and mated them with two three-day old Canton-S males for 24 hours in vials containing yeast glucose food. Six-day old *bam* hypomorph females were subsequently collected for qPCR while the vials were kept at 25°C with a 12-hour light-dark cycle. The progeny from the vials were counted every two days for eight days in total and the sum of the progeny were used for our analyses.

**Statistical Analyses**

To assess correlations between *D. melanogaster* fertility found in Kagemann et al. (2023) and *W. pipientis* variant titer, we used a linear regression model. A linear regression model was conducted in R (v. 4.1.0) at each age/mating status to determine statistically significant differences in *W. pipientis* titer between *D. melanogaster* infected with different *W. pipientis* groups ( *w* Mel-like and *w* MelCS-like) and between the uninfected control (Fig 1A and 1B). The equation used was as follows: *W. pipientis* titer (response variable) ∼ *W. pipientis* Group * Day (Fig 1A and 1B). A two-way ANOVA was used to find differences in *W. pipientis* variant ovary and carcass titer (Fig 1C). To find correlations between *D. melanogaster* fertility and *W. pipientis group* titer in the ovaries and carcass we used a linear regression model (Fig 1D). The equation used was as follows: *D. melanogaster* Fertility ~ *W. pipientis* Group Titer (Fig 1D).
